# Maintaining mitochondrial ribosome function: The role of ribosome rescue and recycling factors

**DOI:** 10.1080/15476286.2021.2015561

**Published:** 2021-12-20

**Authors:** Franziska Nadler, Elena Lavdovskaia, Ricarda Richter-Dennerlein

**Affiliations:** a Department of Cellular Biochemistry, University Medical Center Goettingen, Goettingen, Germany; b Cluster of Excellence “Multiscale Bioimaging: From Molecular Machines to Networks of Excitable Cells” (MBExC), University of Goettingen, Goettingen, Germany

**Keywords:** Mitochondrial ribosome (mitoribosome), translation termination, mitoribosome recycling, mitoribosome rescue, mitoribosome-associated quality control (mtRQC)

## Abstract

The universally conserved process of protein biosynthesis is crucial for maintaining cellular homoeostasis and in eukaryotes, mitochondrial translation is essential for aerobic energy production. Mitochondrial ribosomes (mitoribosomes) are highly specialized to synthesize 13 core subunits of the oxidative phosphorylation (OXPHOS) complexes. Although the mitochondrial translation machinery traces its origin from a bacterial ancestor, it has acquired substantial differences within this endosymbiotic environment. The cycle of mitoribosome function proceeds through the conserved canonical steps of initiation, elongation, termination and mitoribosome recycling. However, when mitoribosomes operate in the context of limited translation factors or on aberrant mRNAs, they can become stalled and activation of rescue mechanisms is required. This review summarizes recent advances in the understanding of protein biosynthesis in mitochondria, focusing especially on the mechanistic and physiological details of translation termination, and mitoribosome recycling and rescue.

## Introduction

Mitochondria are semi-autonomous organelles of eukaryotic cells that maintain a minimal genome (mitochondrial DNA, mtDNA). The organelle was gained as a result of endosymbiosis between an α-proteobacterial cell and a host cell of archaeal lineage [1]. Evolutionary shaping of the mtDNA led to massive depletion of its gene content [2] and as a result, the human mtDNA encodes for just 13 proteins of the OXPHOS system, 2 ribosomal RNAs (rRNA) and 22 tRNAs [3]. However, the mitochondrial gene expression machinery is composed of nuclear-encoded proteins. Defects in components of the mitochondrial translation apparatus lead to OXPHOS dysfunction and contribute to a diverse range of multisystem disorders collectively termed mitochondrial diseases [4–6].

Although the reactions of the translation cycle are conserved among all domains of life, the underlying mechanisms in different systems are adjusted to accommodate specific features of the mRNAs, variations in the genetic code and the peculiarities of synthesized polypeptides [7]. Each step in translation is guided by a conserved set of translation factors, including accessory factors like translational GTPases [8]. In mitochondria, many of these factors have evolved mitochondrial-specific insertions absent from bacterial or cytosolic homologs. Furthermore, certain factors present in other systems are lacking in mitochondria, while others are duplicated and have acquired distinct functions. Evolution of the mitochondrial translation apparatus was tightly coupled with mtDNA reduction and adaptation of the mitochondrial ribosome (mitoribosome) to operate with short, leaderless mRNAs lacking a Shine-Dalgarno (SD) sequence or a cap-like structure [9]. Modern mitoribosomes combine conserved sites involved in mRNA decoding, peptidyl transferase reaction and translation factor association with remodelled regions crucial for mRNA engagement, tRNA binding and nascent polypeptide extrusion [10–13].

The cycle of mitoribosome function consists of four steps: initiation, elongation and termination of the polypeptide, followed by ribosome recycling, which provides free subunits capable of engaging in subsequent rounds of protein synthesis. Translation termination initiates when a stop codon enters the A site of the mitoribosome, where it is recognized by dedicated peptide release factor(s). Formation of the post-termination complex with a deacylated tRNA in the mitoribosomal P site is a pre-requisite of the canonical recycling process. Under certain circumstances, however, mitoribosomes stall on mRNA transcripts and cannot resume protein synthesis. As the biogenesis of mitoribosomes is energetically costly for cells, these trapped complexes must be rescued and recycled to ensure the presence of sufficient mitoribosomes for ongoing translation. The ancestral bacterial system has evolved several strategies to resolve arrested ribosomes [14]; however, these mechanisms are not directly applicable to mitoribosomes. Recent biochemical and structural studies tremendously expanded our knowledge of the mechanisms of translation termination, ribosome recycling and rescue in mitochondria.

In this review, we summarize the recent advances in understanding of mitochondrial translation, highlighting recent discoveries regarding mechanistic details and regulation of each step, guided by nuclear-encoded translation factors. As many aspects of mitochondrial translation remain poorly characterized, we refer to the ancestral bacterial system to appreciate similarities and differences and integrate the available knowledge within the evolutionary context.

## Structural features of the mammalian mitoribosome

The mitoribosome has diverged significantly from its prokaryotic ancestor. In mammals, the fully assembled, mature mitoribosome has a sedimentation coefficient of 55S and a molecular mass of 2.7 MDa. The loss of specific rRNA segments and recruitment of novel proteins during evolution result in a protein-to-rRNA ratio of 2:1, which is the reverse of that observed in ancestral bacterial 70S ribosomes. The mtSSU consists of the 12S rRNA and 30 ribosomal proteins (MRPs) [12,13] whereas the mtLSU encompasses two RNA molecules, 16S rRNA and structural tRNA^Val^ or tRNA^Phe^, and 52 MRPs [10,11]. The structural tRNA is incorporated into the central protuberance (CP) and replaces the otherwise universally conserved 5S rRNA. Mitochondrion-specific elements of the CP (mL40 and mL48) form the P-site finger, which holds the tRNA elbow regions during translation [13,15].

As an adaptation to engage leaderless mRNAs lacking SD sequences, the 3ʹ end of the 12S rRNA does not contain an anti-SD motif and is stably fixed by mS37 at the mRNA exit site, and mRNAs are instead stabilized by interactions with bS1m [12,13]. The remodelled mRNA entrance site consists of a mt-specific extension of uS5m and a pentatricopeptide repeat protein mS39 (PTCD3), which facilitates recruitment of mRNAs to the mitoribosome [12,13].

Nascent polypeptides emerge from the mitoribosome through the polypeptide exit tunnel (PET). The hydrophobic nature of the mitoribosomal PET walls and remodelling of the polypeptide exit site compared to other ribosomes favour the extrusion of membrane proteins, a key feature for expression of the membrane-embedded components of the OXPHOS complexes. When not actively translating, the PET is obstructed by insertion of the N-terminal domain of mL45, which also serves as a membrane anchor [16–18]. When the mitoribosome initiates protein synthesis, the mL45 plug is removed as a result of an interaction with the inner mitochondrial membrane insertase, oxidase assembly 1-like protein (OXA1L) [18,19].

However, despite the changes in rRNA content and overall architecture, the functional centers involved in the universally conserved translation process, are well conserved in the mitoribosome. Thus, the 16S rRNA helices comprising the peptidyl transferase center (PTC) on the mitoribosomal large subunit (mtLSU) as well as the 12S rRNA segments forming the mRNA decoding center on the mitoribosomal small subunit (mtSSU) are similar to those of bacterial 70S ribosomes [10–13]. Like other ribosomes, the mitoribosome bears the functionally conserved tRNA-binding sites: aminoacyl (A) site, peptidyl (P) site and the exit (E) site [12,13,20], and GTPase-associated center (GAC), which includes the L7/L12 stalk, the stalk base and the sarcin-ricin loop (SRL, h95). The GAC recruits translational GTPases and stimulates factor-dependent GTP hydrolysis.

The functional integrity of the mitoribosome relies on intersubunit bridges but the mitoribosomal subunits interact less extensively than those of bacteria, forming only 15 intersubunit bridges [12,13,21]. Remarkably, most of the contacts within mitoribosomes are mediated by protein–protein or protein–RNA interactions, while in bacteria, intersubunit bridges are formed mainly via RNA–RNA contacts [12,13,22].

## The mitochondrial translation cycle

### Initiation

The first step of protein synthesis, initiation, includes mRNA engagement, start codon selection and Watson-Crick pairing with the recruited initiator tRNA. Development of the translationally competent initiation complex (IC) proceeds through the formation of several intermediates. In mitochondria, initiation requires the assistance of only two initiation factors: mtIF2 and mtIF3. Lack of IF1 in mitochondria is compensated by the mitochondrion-specific domain insertion within mtIF2, which prevents the A site from premature tRNA accommodation during IC formation [17,23,24].

**Figure 1.**
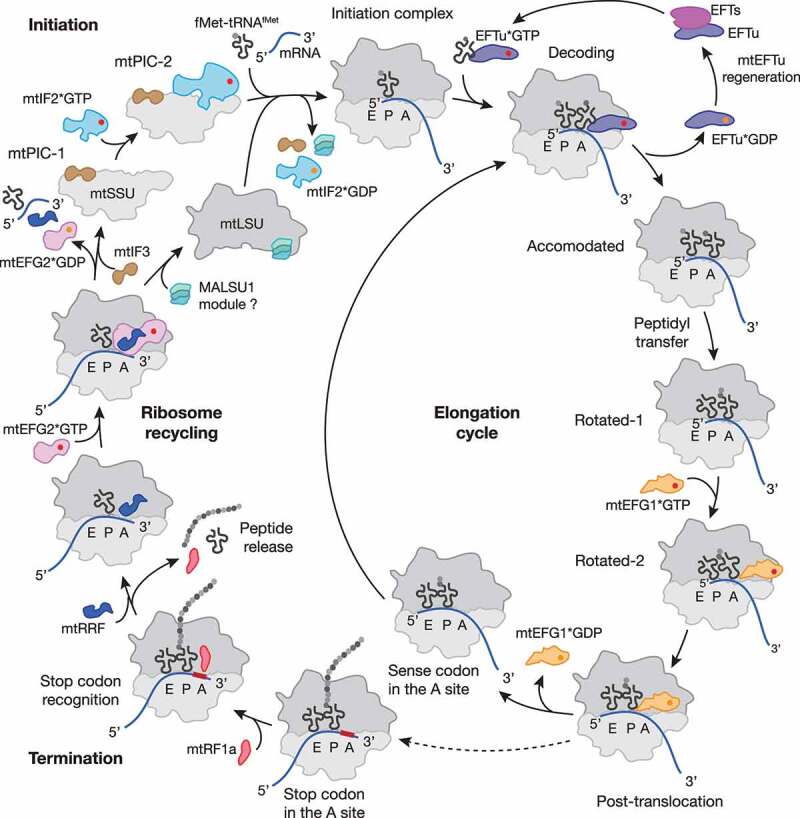
**Schematic representation of the mitochondrial translation cycle**. Mitochondrial protein synthesis follows the four steps of translation initiation, elongation, termination and ribosome recycling (for details see main text). Translation factors are depicted as follows: mtIF2, light blue; mtIF3, brown; mtEFTu, purple; mtEFTs, magenta; mtEFG1, Orange; mtRF1a, red; mtRRF, dark blue; mtEFG2, pink; MALSU1 module, turquoise; GTP and GDP are shown as red and Orange circle, respectively. Most of the depicted states were structurally resolved by cryo-EM (for PDB codes and further details, see Table 1). The MALSU1 module composed of MALSU1, L0R8F8 and mtACP may act as an anti-association factor during ribosome recycling.

The first complex, designated as pre-initiation complex 1 (mtPIC1), consists of the mtSSU and initiation factor 3 (mtIF3) (Figure 1, Table 1) [25–27]. mtIF3 forms extensive contacts with the mtSSU platform, thereby preventing the complex from premature association with the mtLSU and initiator fMet-tRNA^fMet^ [26,27]. Next, interaction of the mtIF3 N-terminal domain (NTD) with the mitochondrion-specific protein mS37 at the mRNA channel exit compresses h28 of the 12S rRNA at the mtSSU neck and allows mtIF2*GTP recruitment, resulting in formation of mtPIC2 [27]. The following events can likely proceed via several alternative pathways [28]. The first scenario is reminiscent of bacteria and would involve mtIF2-mediated recruitment of the initiator fMet-tRNA^fMet^, while mtIF3 ensures the correct codon-anticodon pairing between mRNA and fMet-tRNA^fMet^ in the P site [17,25,26,29–31]. However, *in vitro* reconstitution of the mitochondrial ICs and subsequent structural analysis suggest that, in contrast to translation initiation in bacteria, recruitment of the mRNA and fMet-tRNA^fMet^ occurs concurrently with subunit joining and strictly require prior dissociation of mtIF3 from the complex [27]. The second envisaged scenario is reminiscent of leaderless mRNA loading onto assembled bacterial 70S ribosome complexes, where IF3 occupies a non-canonical position on the 50S subunit to perform its function in initiation complex formation [32,33].

**Table 1.** Cryo-EM structures of mitochondrial translation and ribosome rescue states

Structure	PDB/EMD code	Description	Ref.
mtPIC1	PDB 6RW4	28S + mtIF3	^[27]^
mtPIC1	PDB 6NF8 PDB 6NEQ	28S + mtIF3	^[45]^
mtPIC2* (w/o mtIF3)	PDB 6GAZ	28S + mtIF2	^[17]^
mtPIC2	PDB 6RW5	28S + mtIF3 + mtIF2	^[27]^
55S-IC	PDB 6GAW	55S + mtIF2 + mRNA + fMet-tRNA^fMet^ (P site)	^[17]^
Decoding complex	PDB 7A5G	55S + A/T-tRNA/mtEFTu + P/P tRNA/nascent polypeptide + E/E-tRNA	^[19]^
Accommodated complex	PDB 7A5I	55S + A-tRNA + P-tRNA/nascent polypeptide + E-tRNA	^[19]^
Accommodated complex	PDB 6ZM5	55S + A/A-tRNA + P/P-tRNA/nascent polypeptide + OXA1L	^[18]^
Accommodated complex	PDB 6ZSG	55S + A/A-tRNA + P/P-tRNA + E-tRNA	^[15]^
Rotated-1 complex	EMD-11636	55S + A/A-tRNA/nascent polypeptide + P/E-tRNA	^[19]^
Ti Post-translocation (Rotated-2) complex	PDB 6YDW	55S + A/P-tRNA/nascent polypeptide + P/E-tRNA + mtEFG1*(GDP+Pi)	^[48]^
Rotated-2 complex	PDB 6VLZ	Poorly resolved: 55S/ rotated mtSSU + E-tRNA + mtEFG1*(GMPPCP)	^[49]^
Rotated-2 complex	PDB 6ZSE	55S + A/P-tRNA + P/E-tRNA	^[15]^
Post-translocation complex	PDB 6VMI	55S + P-tRNA + E-tRNA + mtEFG1*(GMPPCP)	^[49]^
Post-translocation + Vacant A-site complex	PDB 6YDP	55S + P-tRNA/nascent polypeptide + mtEFG1*(GDP+Pi)	^[48]^
Post-translocation complex	PDB 7A5K	55S + P-tRNA/nascent polypeptide + E-tRNA + mtEFG1*(GMPPCP)	^[19]^
Vacant A-site* (transient state)	PDB 7A5F	55S + P-tRNA/nascent polypeptide + E-tRNA	^[19]^
Termination complex	PDB 7NQH	55S + P-tRNA + mtRF1a	^[47]^
Recycling complex-1	PDB 6ZS9	55S + mtRRF	^[15]^
Recycling complex-1	PDB 7NSI	55S + P/E-tRNA + mtRRF	^[47]^
Recycling complex-1	PDB 6NU2 PDB 7L08	55S + P/E-tRNA + mtRRF	^[45,46]^
Recycling complex-2	EMD-23114	55S + E-tRNA + mtRRF + mtEFG2*(GMPPCP)	^[46]^
Split mtLSU (canonical recycling)	PDB 7NSH	39S + mtRRF + mtEFG2*(GDPNP)	^[47]^
Split mtLSU (canonical recycling)	PDB 7L20	39S + E-tRNA + mtRRF + mtEFG2*(GMPPCP)	^[46]^
Non-stop rescue complex	PDB 7NQL	55S + P-tRNA + ICT1	^[47]^
Split mtLSU (alternative recycling)	PDB 7OF4 PDB 7OF6 (distinct PTC conformations)	39S + GTPBP6*(GTP)	^[133]^
Split mtLSU (mtRQC)	PDB 7A5H	39S + P-tRNA/nascent polypeptide + mtRF-R + MTRES1	^[19]^

The precise function of mtIF3 in mitochondrial translation initiation remains controversial. Biochemical studies in a yeast model have demonstrated that mtIF3/Aim3 depletion leads to translation imbalance resulting in decreased levels of COX1 and COX2, and increased levels of ATP6 and ATP9 [34]. In line with these observations, ablation of mtIF3 in cultured cells results in a significant decrease of ATP6 (translated downstream of ATP8) and possibly ND4 (downstream of ND4L) [35]. In contrast, ablation of mtIF3 in mouse is embryonic lethal, and conditional heart- and skeletal muscle–specific knock-out leads to imbalanced translation [31]. Importantly, in the same study, mitoribosome profiling revealed an increase in ATP6/8, ND4/4 L and ND5 footprints in the knock-out cells [31]. Thus, loss of mtIF3 specifically affects the translation of the two mitochondrial bicistronic transcripts, likely due to mitoribosome stalling on these mRNAs [31,35]. It is tempting to speculate that the factor fulfils an essential role in bicistronic transcript translation [8]. Although the exact mechanism of ATP6/8 and ND4/4 L translation remains unknown, a recent study by Cruz-Zaragoza et al. (2021) demonstrated that *ATP8* translation is a prerequisite for *ATP6* translation and that *ND4* translation depends on *ND4L* translation [36]. Whether mtIF3 plays an active role in this interdependent process, which would require a backward movement of the ribosome and reinitiation, needs to be further addressed.

Another long-standing question concerning mRNA loading onto mitoribosomes has recently been solved using single particle cryogenic electron microscopy (cryo-EM). As already mentioned, mitochondrial mRNAs (mt-mRNAs) lack an SD sequence or a cap structure at the 5ʹ end, and therefore, their recruitment to mitoribosomes occurs via a mechanism distinct from bacteria and eukaryotes. The delivery of mt-mRNAs to the mRNA entry channel is mediated by the LRPPRC/SLIRP complex, which binds to the pentatricopeptide repeat (PPR) domain-containing mitoribosomal protein mS39 [15,17]. After the IC is formed, contact between mtIF2 and the SRL facilitates GTP hydrolysis by mtIF2, and the factors leave the assembled complex, allowing transition to the elongation phase.

### Elongation

During elongation, the ribosome translocates along the mRNA transcript, converting the genetic information into the amino acid sequence of a polypeptide. The process proceeds through three cycles: mRNA codon decoding by a cognate tRNA, peptide bond formation and translocation of the mRNA–tRNA module (Figure 1, Table 1). The basic mechanism of elongation remains conserved among all ribosomes [8,29,37].

Transition from initiation to the elongation phase is coupled with the delivery of the second aa-tRNA (aminoacyl tRNA) to the mitoribosomal A site by the mtEFTu*GTP complex (elongation factor thermo unstable) [38,39]. Evolutionarily conserved rRNA helices of the mtSSU decoding center proofread the basepairing between the mRNA codon and the anti-codon of the newly delivered tRNA to ensure cognate aa-tRNA binding [19]. If the quality control criteria are fulfiled, mtEFTu*GTP comes into close contact with the SRL, which mediates GTP hydrolysis and subsequent mtEFTu*GDP release from the translation complex [19,40]. Re-charging of mtEFTu with GTP is performed by mtEFTs (elongation factor thermo stable), another conserved factor [41,42].

The correct accommodation of the aa-tRNA in the A site allows the second elongation step, the peptidyl transferase reaction. This reaction is catalysed by the conserved PTC of the mitoribosome, which comprises solely elements of the 16S rRNA and facilitates the transfer of the nascent polypeptide from the P site tRNA to the α-amine of the A site tRNA [10,11]. The final step of the elongation process, namely translocation, involves the high-fidelity in-frame movement of the mRNA with bound tRNA by exactly three nucleotides. During this process, the deacetylated P site tRNA and A site tRNA bearing the polypeptide chain shift to the E and P site respectively. The E site tRNA is then ejected by conserved elements of the L1 stalk. This process is coupled with multiple large-scale movements of the mtSSU and proceeds through the formation of tRNA hybrid states [19]. In bacteria, translocation is catalysed by EFG*GTP (elongation factor G), which stabilizes the hybrid states and facilitates translocation [43]. Mitochondria have evolved two homologs of the bacterial elongation factor: mtEFG1 and mtEFG2. While mtEFG1 is required for translation elongation, mtEFG2 specifically acts in concert with the ribosome recycling factor mtRRF, initiating splitting of the mitoribosomal subunits at the end of the translation cycle [44–47]. The function of mtEFG1 is reminiscent of bacterial EFG and coupled with GTP hydrolysis induced by the mitoribosome GAC [48,49]. This factor prevents retrieval of the P site tRNA back to the A site and facilitates mtSSU rotation, thus driving the hybrid tRNAs to adopt their new positions [19,48,49]. When the translocation step is accomplished, mtEFG1*GDP leaves the translation complex and a new mRNA codon is present in the A site ready for the next cycle of elongation [48,49].

### Translation Termination

When the ribosome translocates to a mRNA codon not assigned to a cognate tRNA, the final stage of protein synthesis, translation termination, occurs. The stop codon is detected by release factors, which trigger hydrolysis of the polypeptide chain from the peptidyl-tRNA.

Translation termination in bacteria

The decoding mechanism responsible for recognizing the termination codon and subsequent cleavage of the polypeptide chain from the peptidyl-tRNA on translating 70S ribosomes requires a release factor, which specifically recognizes and binds to the translation termination codons UAA, UAG and UGA in a codon-dependent manner [50]. Codon recognition is performed by two distinct release factors: RF1 and RF2, which are responsible for the recognition of UAA and UAG or UAA and UGA codons, respectively [51,52].

The prokaryotic release factors harbour a recognition motif that resembles a tRNA anticodon, thereby endowing them with codon specificity. This motif consists of a tripeptide, which is either proline-x-threonine (PxT) for RF1 or serine-proline-phenylalanine (SPF) for RF2 [53]. With emergence of crystal and cryo-EM structures of RF1 and RF2 bound to the ribosome, the precise mechanism of decoding was solved [54–57]. These studies demonstrated that not only the recognition motif interacts directly with the stop codon but that also the tip of α5 helix plays an important role. Together, those two elements act as ‘molecular tweezers’; upon entering the decoding site of a ribosome and sensing a stop codon, the release factor interacts with the stop codon with high affinity, inducing conformational changes in the ‘closed’ release factor, triggering it to adopt an open conformation and enabling domain 3 to reach the PTC. A sense codon on the other hand is recognized with low affinity and thereby the subsequent conformational change in the release factor is not provoked. This mechanism prevents premature termination while also enabling efficient translation by coupling codon recognition and peptide release [58,59].

Promoting hydrolysis of the ester bond between the nascent polypeptide chain and the peptidyl tRNA is mediated by the highly conserved GGQ motif in domain 3. This amino acid sequence, present in all known release factors in the three domains of life, is essential for proper release factor function as it stimulates the peptidyl-tRNA hydrolase (PTH) activity [60,61].

The distance between the decoding center of the 30S SSU and the PTC of the 50S LSU is approximately 75 Å whereas the distance between the decoding motif and the GGQ motif of the release factor is only 23 Å. The overall structure of the release factor therefore needs to elongate to span these features. If a stop codon is sensed correctly, the release factor is tightly associated with the stop codon and dependent on this event, conformational changes in the so-called ‘switch-loop’ are induced. The switch-loop connects the GGQ-harbouring domain 3 and domain 4 and upon its extension, domain 3 reaches into the PTC where it interacts with the peptidyl-tRNA. The open conformation of the release factors is reminiscent of the overall shape of a tRNA and the GGQ motif mimics the typical CCA extension. Once present in the PTC, the two glycine residues of the GGQ motif may form a channel providing access for the water molecule needed for the nucleophilic attack, thus enabling the peptide to be hydrolysed and released [54,55,57].

Mitochondrial translation termination

In human mitochondria, the genetic code differs from the universal one. While the three codons UGA, UAG and UAA are specified as stop codons in bacteria and the eukaryotic cytosol, UGA is reassigned as to code for tryptophan in human mitochondria [62]. Interestingly, two alternative stop codons terminate translation of the mt-mRNAs *MT-CO1* (mRNA encoding for COX1) and *MT-ND6* (mRNA encoding for ND6); there are no complementary tRNAs for the AGA and AGG codons in human mitochondria, implying that they function as stop codons and not as arginine codons like in bacteria or the cytosol [63]. Whether the two proposed additional stop codons AGA and AGG function as alternative termination codons in mitochondria and whether they are recognized by dedicated release factors is still under debate. A − 1 frameshift mechanism has been suggested for *MT-CO1* and *MT-ND6*, which would lead to termination at a conventional UAG stop codon as the preceding nucleotide is a uridine in both cases [64].

Based on their homology to the bacterial release factors, four putative mitochondrial release factors have been identified: mtRF1, mtRF1a, ICT1/mL62 and C12ORF65. While all four factors contain a GGQ motif-containing PTH domain, only mtRF1 and mtRF1a also contain a codon recognition domain. In contrast, ICT1 and C12ORF65 possess positively charged C-terminal extensions of different lengths, which are similar to those found in bacterial rescue factors.

Which of the two codon-specific release factors is the main mitochondrial release factor was, for a long time, an outstanding question. Although mtRF1 was identified as the first mammalian mitochondrial release factors decades ago [65,66], an increasing body of evidence supported the role of mtRF1a as the canonical release factor in mitochondria [47,64,67–70].

The most striking difference between mtRF1 and mtRF1a resides in their codon recognition domain. While mtRF1a shows high similarities to *E.coli* RF1 in the decoding motifs, having a classical tripeptide motif (PKT) and α5 helix tip, two extensions are present in the corresponding motifs of mtRF1: a two amino acid (RT) insertion just before the tip of the α5 helix and an extended codon recognition hexapeptide motif (PEVGLS). Whether these extensions represent co-evolved features that allow mtRF1 to accommodate changes in mt-rRNA structure and the overall mitoribosomal architecture remains elusive [20,67,70].

The function of mtRF1 remains largely unknown as despite being a mitochondrial protein with high sequence homology to bacterial class I release factors, mtRF1 does not show any release factor activity *in vitro* using bacterial 70S ribosomes irrespective of which codons were tested [68,69]. As *in silico* studies and computational data come to different conclusions, there is dissent regarding the function of mtRF1. Homology modelling and hybrid activity assays where domains critical for codon recognition by bacterial RF1 were exchanged with the cognate domains of mtRF1 suggest that mtRF1 may be a potential candidate for decoding the non-standard stop codons AGA and AGG as the large adenine purine base could be accommodated by its extended decoding motifs [70]. However, this possibility was ruled out by Lind et al. (2013) and Huynen et al. (2013) [67,71]. Based on homology modelling, molecular dynamics and free-energy calculations, Lind and colleagues proposed that mtRF1 has the same codon reading qualities as bacterial RF1 and mtRF1a, and thus could recognize canonical UAG and UAA as stop codons. Huynen and co-workers suggested that mtRF1 might instead be involved in ribosome rescue by binding ribosomes with an empty A site lacking mRNA as it would otherwise sterically clash with a stop codon accommodated in the ribosomal A site. However, a recent structural attempt failed to recover mitoribosomes with mtRF1 bound in positions supporting any of these scenarios, leaving the question of its role in translation termination still open [47].

In contrast, a clear consensus regarding the function of mtRF1a has emerged. In line with the high similarity to bacterial RF1, mtRF1a shows the same codon reading abilities on canonical UAG and UAA stop codons and also displays PTH activity when biochemically assessed in *in vitro* translation termination assays [68,69]. mtRF1a could potentially terminate translation of all 13 mitochondrial-encoded transcripts, as 11 are directly terminated by UAG or UAA codons and termination of *MT-CO1* and *MT-ND6*, which are supposed to be terminated by AGA or AGG, also would present an UAG stop codon upon −1 ribosome frameshifting.

Recent structural data of mtRF1a bound to mitoribosomes programmed with either UAG- or UAA-containing mRNAs confirmed the role of mtRF1a in translation termination upon recognition of standard stop codons (Figure 1, Table 1) [47]. Interactions observed between bacterial RF1, the ribosome and the mRNA are highly conserved in the mtRF1a complex [55]. The codon recognition domain is positioned in the decoding center and deciphers mRNA codons in the same way as its bacterial counterpart; while the first position U is coordinated by Thr208 (Thr186 in RF1) of the PxT motif and the tip of the α5 helix, the A at the second position is also in close contact with Thr208 as well as other adjacent residues. Similar to bacterial RF1, the third position nucleotide is stacked against G256 (bacterial G530) of the mitoribosomal 12S rRNA and similarly contacted by two residues of mtRF1a (Thr216 and Glu203) that would accommodate either an A or G at this position. Upon binding, the switch-loop of mtRF1a undergoes large conformational changes so that the catalytically active PTH domain can reach the PTC, stabilized via stacking interactions similar to its bacterial homolog, and thus trigger peptide hydrolysis.

### Ribosome recycling

After translation termination, the ribosome undergoes a recycling step, which includes disassembling of the post-termination ribosome complex (PoTC) into ribosomal subunits and ejection of the remaining tRNA and mRNA. In bacteria and mitochondria, recycling is carried out by three factors: ribosome recycling factor ((mt)RRF), EFG/mtEFG2, and (mt)IF3 (Figure 1, Table 1) [8,29]. The general mechanism of ribosome recycling seems to be conserved from bacteria and involves the collaborative action of RRF and EFG to disassemble the PoTC. To prevent subunit re-association, IF3 binds to the SSU blocking elements involved in intersubunit bridge formation and facilitating release of the remaining mRNA [29,30]. Similarly to mtIF3 acting as an anti-association factor bound to the mtSSU, the MALSU1-module (composed of MALSU1, L0R8F8, and mtACP) was recently shown to be bound to split mtLSU complexes, suggesting dual functions during ribosome biogenesis and recycling as discussed later [19] (Figure 1, Table 1).

The central player of the canonical recycling pathway, (mt)RRF, retains a conserved, essential function from eubacteria to human mitochondria [72,73]. The factor consists of triple helix bundle in domain I, connected via flexible linkers to domain II [45–47]. In addition, human mtRRF contains an N-terminal extension that is not cleaved upon protein import into mitochondria [73]. The precise function of the extension is controversially discussed. A study by Koripella et al. (2021) [46] suggested the extension to be important for stabilizing the rotated conformation of the 55S complex, precluding mtEFG2 binding. However, in a parallel study by Kummer et al. (2021) [47], an N-terminally truncated isoform of mtRRF was used and the recycling efficiency was unaffected.

EFG and its mitochondrial homologs mtEFG1 and mtEFG2 are translational GTPases consisting of five highly conserved domains [43,74]. These domains are folded into two super-domains (domain I+ II and domain III–V) that are connected via a flexible linker [46–49,75]. The presence of two paralogs in human mitochondria implies the existence of a mechanism to ensure binding of the appropriate factor to elongating ribosomes (mtEFG1) or to PoTCs (mtEFG2). mtEFG2 is compositionally and functionally specialized for recycling; firstly, in contrast to mtEFG1, its binding to the mitoribosome complex depends on the presence of mtRRF and secondly, mtEFG1 has a C-terminal extension and positively charged amino acid patch, which preclude its interaction with mtRRF [46–49].

The third factor, (mt)IF3, is not directly involved in ribosome dissociation but instead, prevents subunit re-association by blocking the SSU [76]. Thus, (mt)IF3 factor possesses dual functions by acting in both translation initiation and ribosome recycling. However, a recent study by Rudler et al. (2019) [31] suggested that the anti-association function might be dispensable in human mitochondria.

A prerequisite of the canonical recycling pathways in both bacteria and mitochondria is the PoTC with an empty A site and non-charged tRNAs in the P and E sites [8,29]. This status is of high physiological importance as it ensures that only ribosomes which have terminated protein synthesis can enter the recycling step. The mechanism of discriminating ribosomes with this status has been elucidated through structural studies, which demonstrated that binding of (mt)RRF facilitates the P site tRNA to shift into P/E state whereupon domain I of (mt)RRF blocks the PTC from tRNA engagement [45,46,77,78]. This movement would only be achievable for a deacylated tRNA, otherwise the remaining polypeptide would make it sterically impossible.

Binding of mtRRF to the 55S mitoribosome stabilizes the rotated state of the mtSSU [15,45–47]. In contrast, bacterial RRF is able to associate with both rotated and non-rotated 70S PoTC [78–80]. The overall position of mtRRF on the 55S PoTC complex is reminiscent of bacteria with the triple helix bundle of domain I embracing the A and P sites formed by 16S rRNA helices [45–47]. Accommodation of mtRRF on the fully rotated 55S PoTC destabilizes seven of the 15 inter-subunit bridges [46]. In bacteria, EFG follows the same mode of action during translation elongation and ribosome recycling. EFG*GTP binding to the ribosome complex does not require the presence of RRF and occurs in a compact conformation [43]. To facilitate mRNA-tRNA translocation or PoTC dissociation, EFG adopts an extended conformation and its GTPase domain interacts with the SRL. As a result of GTP hydrolysis during this translocation step, EFG facilitates SSU rotation and mRNA-tRNA movement through the ribosome [43]. In case of recycling, when RRF is present, the extended domains III and V of EFG create a cleft with embedded domain II of RRF [78]. The motion induces RRF domain II to rotate towards helix 69 of the 23S rRNA. Cooperative movement of helix 69 and domain II of RRF towards the B2a intersubunit bridge causes its disruption [78,80]. Similarly, the mitochondrial recycling machinery extracts the critical intersubunit bridge-forming helix 69 of the 16S rRNA by capturing it between domains I and II of mtRRF in a tweezer-like manner and directly lifting it away from helix 44 [46,47]. In case of mitoribosome recycling, this motion is induced by mtEFG2*GTP binding, and, in contrast to bacteria, is not coupled to GTP hydrolysis [44,46,47]. The helix displacement by mtRRF leads to disruption of the central B2a intersubunit bridge [46,47] and probably also the B3 intersubunit bridge [46].

## What can go wrong: ribosome pausing vs. stalling

The velocity of translation is not linear and differs between various mRNAs as well as across distinct regions of a given mRNA. Rapid protein synthesis alternates with ribosome deceleration or pausing. Ribosome pausing in bacteria usually results from the mRNA primary or secondary structural features, such as the presence of rare codons, poly-Lys or poly-Pro stretches encoded within the message, or aminoacyl tRNA starvation [81]. It serves as a key mechanism to adjust protein synthesis in changing environmental conditions and to ensure the fidelity of the cellular proteome. Paused ribosomes are able to resume translation when the underlying problem is solved, for example, when tRNAs become available again or the challenging nucleotide cluster has been passed. Although the dynamics of mitoribosome movements along mt-mRNAs currently remain unexplored, accumulating evidence suggests that mitoribosome pausing is required to coordinate the production of the mtDNA-encoded OXPHOS subunits with the influx of the nuclear-encoded subunits [82,83].

Under certain circumstances, however, arrested ribosomes are not capable of resuming translation and the intervention of rescue mechanisms is required. The main causes of ribosome stalling are aberrant mRNAs, defective translation factors or errors within the ribosome itself [84,85]. For example, in mitochondria, aberrant mRNAs can arise as a result of partial nucleolytic degradation or incorrect processing, or defects in polyadenylation, which lead to incomplete stop codon in certain ORFs [8,9].

As co-transcriptional mRNA quality-control mechanisms are absent in bacteria and mitochondria, their ribosomes can engage and consequently initiate translation of aberrant mRNAs. As a result, ribosomes not able to terminate correctly are therefore stalled at the 3ʹ end of truncated mRNAs due to the lack of a codon in the ribosomal A site. Bacteria have evolved different pathways to resolve such stalled translation complexes: the *trans*-translation system, the alternative rescue pathways initiated by ArfA/ArfB and the RqcH-mediated ribosome-associated quality control mechanism [84–86]. These mechanisms are invoked to overcome ribosome stalling and thereby recycle ribosomal subunits to prevent diminished protein synthesis capacity.

## Ribosome rescue pathways in bacteria

### Trans*-translation system*

The most critical form of ribosome stalling happens when a so-called ‘non-stop’ translation complex is formed due to the lack of an mRNA codon in the decoding center In this case, the ribosome is trapped in a complex where it is tightly bound to the mRNA and the peptidyl-tRNA. Neither elongation factors nor a canonical release factor can act on these stalled ribosomes and the peptidyl-tRNA in the P site prevents binding of RRF thus precluding dissociation of the ribosome into its subunits [87]. *Trans*-translation was identified as the main bacterial rescue mechanism as genes encoding at least one of the core components have been found in almost all bacteria [88]. The central components of this rescue system are the transfer-messenger RNA (tmRNA, encoded by *ssrA*) and the RNA-binding protein SmpB [89,90].

The tmRNA is a specialized RNA with properties of both tRNA and mRNA as it contains a tRNA-like domain (TLD) lacking an anticodon region but with an acceptor arm and an internal open reading frame that serves as an mRNA-like template. The 3ʹ end of the tmRNA is recognized by the canonical alanyl-tRNA synthetase (AlaRS) and can thereby be charged with alanine. The interaction between tmRNA and AlaRS is further increased by the binding of SmpB to the TLD. This complex mimics a classical tRNA as the globular domain of SmpB replaces the anticodon stem loop of the tRNA. Upon binding of the canonical elongation factor EFTu to the TLD, the tmRNA-SmpB complex is delivered to the A site of non-stop stalled ribosomes. Interference with elongating ribosomes is prevented by the C-terminal tail of SmpB, which adopts an α-helical conformation when bound to the ribosome and thereby senses whether the mRNA channel is empty. Upon entering the ribosomal A site, the acceptor arm of tmRNA is placed in the PTC such that the truncated, nascent polypeptide chain can be transferred to the tmRNA-alanine and binding of EFG then enable the complex to translocate to the P site. Consequently, the tmRNA is first in an A/P hybrid-state and large rotational conformation changes of the 30S head allow the mRNA-reminiscent part of tmRNA to be placed in the mRNA channel, thereby displacing the C-terminus of SmpB. Upon translocation of the first codon (the resume codon), the internal open reading frame is placed at the A site. It encodes for a short protein tag (AANDENYALAA), which marks aberrant proteins for degradation. Elongation continues as usual until the stop codon is reached and translation is terminated by canonical release factors. The tagged polypeptide is released, recognized by several proteases and consequently degraded [90–92]. *Trans*-translation also serves as mRNA quality control mechanism by recruiting RNase R to degrade the aberrant mRNA, thereby preventing re-initiation of translation on dysfunctional mRNAs [93]. Via this process, the ribosome can also be released and recycled for another translation cycle.

### Alternative ribosome rescue pathways

#### ArfA

Although *trans*-translation appears to be the major bacterial ribosome rescue pathway, mutations in the *ssrA* gene leading to depletion of tmRNA are not lethal in most bacteria, indicating that alternative rescue pathways can compensate for the loss of *trans*-translation. Mutational screening in *E. coli* identified YhdL, renamed ArfA (alternative ribosome-rescue factor A), as the protein required for cell viability in the absence of tmRNA-based *trans*-translation. Lack of both *trans*-translation and ArfA is lethal in *E. coli*, confirming the physiological importance of ribosome rescue mechanisms [94]. As ArfA does not exert any PTH activity on its own, it requires an additional factor to release ribosomes stalled on truncated mRNAs. ArfA binds at a vacant ribosomal A site and recruitment of the canonical release factor RF2 induces hydrolysis of the aberrant peptide from the P site tRNA. ArfA serves as an adapter molecule for RF2, as its codon recognition domain (SPF) is not required for binding to the ArfA-non-stop ribosome complex. The conserved GGQ domain of RF2 is, however, essential for ArfA-mediated ribosome rescue as it triggers hydrolysis of the peptidyl tRNA in the PTC [95,96]. Mechanistically, ArfA probes for the presence of an mRNA as already shown for tmRNA-SmpB [97,98]. By inserting its highly conserved, positively charged C-terminus from the A site of the decoding center into the mRNA channel formed by the negatively charged 16S rRNA, ArfA is anchored at the intersubunit region by electrostatic interactions.

Notably, in contrast to tmRNA-based *trans*-translation, the ArfA-based rescue mechanism does not add a degradation signal to proteins released from non-stop ribosomes to mark them for proteolytic cleavage and thus does not contribute to protein quality control. Moreover, bacteria have developed a sophisticated mechanism to tightly coordinate ArfA expression and the need for ArfA-mediated ribosome rescue [99,100]. Transcription of *arfA* results in an mRNA containing hairpin structure, which is recognized and cleaved by RNAse III, thereby generating a truncated *arfA* mRNA lacking a stop codon. Consequently, a non-stop complex is formed when translating the *arfA* mRNA, and this is targeted by *trans*-translation thus tagging ArfA for subsequent proteolysis. However, if tmRNA activity is limited, disabled or overwhelmed, an active form of ArfA is translated, which can fulfil its function as a ribosome rescue factor thereby serving as a backup rescue system. In this way, unnecessary competition between the two rescue mechanisms is prevented by keeping the level of ArfA minimal as long as tmRNA is sufficient to not only detect but also ultimately degrade non-stop complexes.

#### ArfB

A second alternative rescue system present in a wide range of bacteria can also release ribosomes stalled at non-stop complexes [101]. The *E. coli* YaeJ protein is able to facilitate peptide hydrolysis in the absence of an mRNA in the ribosomal A site and was therefore renamed as ArfB. In *E. coli*, although the chromosomally encoded copy of ArfB cannot rescue the lethal phenotype of the double *arfA ssrA* mutant, ArfB can act as a multicopy suppressor. In contrast to ArfA, ArfB possesses an N-terminal domain containing a GGQ motif structurally and functionally similar to domain 3 present in the canonical release factors and is thereby implicated in PTH activity. However, instead of a codon recognition domain, similar to SmpB and ArfA, ArfB has a C-terminal domain consisting of basic residues, which is important for ribosome binding [102,103]. Indeed, the C-terminal tail of ArfB exerts the same function as SmpB and ArfA by forming an α-helical element with its positively charged residues and thereby sensing the occupancy of the mRNA channel to distinguish between actively translating ribosomes and those that are stalled due to translation of truncated mRNAs. Analogous stacking interactions as in the canonical decoding scenarios take place that not only anchor the protein at the decoding center of the small subunits but also induce conformational changes in the linker region of the protein so that the N-terminal globular domain can position its GGQ loop in close proximity to the CCA end of the peptidyl tRNA and thus trigger hydrolysis of the ester bond of the nascent polypeptide [104,105]. Consequently, there are several highly conserved residues in ArfB homologs not only in the GGQ domain but also at the C-terminal tail and the linker region, which are responsible for effective ribosome-binding and thus PTH activity [106]. Similar to ArfA, ArfB does not tag the released polypeptide for proteolytic degradation.

It still needs to be elucidated, however, whether the physiological function of ArfB needs specific (stress) conditions that favour the high copy expression of ArfB and limit the expression of tmRNA and ArfA. Thus, it is plausible that ArfB is not another backup rescue mechanism if *trans*-translation and/or ArfA are disabled, but rather could act in other possible pathways [87]. For example, ArfB was suggested to cooperate with a stress-induced ribosome recycling factor HflX (high-frequency lysogeny, locus X) [107]. HflX expression is typically nearly non-detectable under physiological conditions, but dramatically increases upon heat-shock, antibiotic treatment or manganese stress [108–110]. In this situation, ArfB facilitates nascent peptide release from the arrested ribosome, allowing HflX to enter the complex. Binding of HflX induces disruption of the B2a intersubunit bridge, thereby initiating ribosome dissociation [107]. Importantly, HflX remains bound to the LSU, preventing its engagement into translation until potential damages are eliminated [109,111].

### RqcH/RqcP: ribosome-associated quality control in bacteria

Recently, a novel ribosome-associated quality control (RQC) mechanism was described in *Bacillus subtilis* [86]. The pathway is mediated by RqcH, a protein homologous to eukaryotic Rqc2/NEMF. To initiate the process, the ribosomal subunits must separate during translation elongation without release of the nascent polypeptide from the tRNA bound to the LSU. In eukaryotes, such ribosome dissociation is executed by Hbs1/HBS1L and Dom34/Pelota with an assistance of ABCE1 [112], but the underlying ribosome dissociation mechanism in bacteria remains unsolved [14]. Remarkably, re-association of ribosomal subunits undergoing the RQC is prevented by RsfS, the bacterial homolog of human MALSU1, bound to the split LSU [113,114].

Structural analyses allowed visualization of the post-splitting LSU complex with RqcH embracing the L7/L12 stalk, the CP and the PTC, and a peptidyl tRNA bound in the P site [113,115]. RqcH requires the assistance of another factor, RqcP/YabO, to resolve the aborted LSU complex. The suggested mechanism of RqcH-mediated RQC [113,115] implies the binding of RqcP to the peptidyl tRNA and its stabilization in the P site. Next, RqcH delivers the Ala-tRNA^Ala^ to the A site, mimicking the action of EFTu during conventional translation elongation. After Ala-tRNA^Ala^ enters the A site, peptidyl transfer is facilitated. The C-terminal Ala tailing marks the aberrant polypeptide for degradation in both mammalian cytosolic and bacterial RQC pathways [14,112]. To allow the P site tRNA to relocate to the E site and eventually leave the LSU, RqcP temporarily dissociates from the complex. Apparently, when the tRNA is positioned in the E site, RqcP can re-bind and tRNA^Ala^ bearing the nascent polypeptide shifts to the P site. The RQC poly-Ala elongation cycle, mediated by the concerted action of RqcH/RqcP, repeats until an unknown factor(s) terminates the process. In the equivalent eukaryotic system, the cycle is aborted by the action of Vms1/ANKZF1 [116,117]. After the polypeptide chain is extracted, the C-terminal alanine tail is recognized by the protease ClpXP [86].

In the eukaryotic cytosol, RQC is initiated in response to ribosomes arrested in the scenarios of non-stop translation, tRNA starvation or when ribosomes collide on mRNAs [85,112]. The physiological relevance of the RqcH/RqcP-mediated RQC in bacteria is currently unknown but it was suggested to be induced by translation elongation stalling due to heat stress or antibiotic treatment [86].

## Ribosome rescue in human mitochondria

### ICT1/mL62

**Figure 2.**
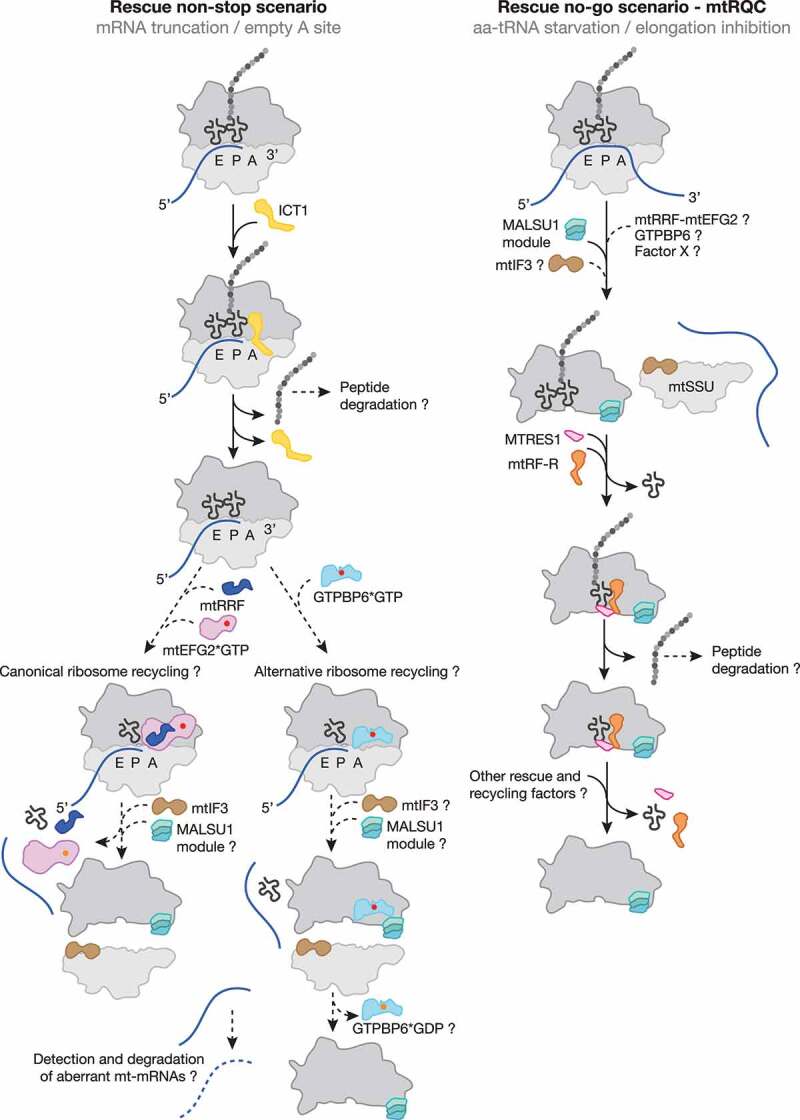
**Mitochondrial ribosome rescue**. Two pathways have recently been described involving two members of the release factor family, ICT1 (yellow) and C12ORF65 (renamed to mtRF-R, mitochondrial release factor in rescue; Orange). ICT1 targets stalled ribosome complexes with an empty A site due to truncated mRNAs (non-stop rescue pathway). Whether ribosomes follow the canonical (mtRRF, dark blue; mtEFG2, pink) or the alternative recycling pathway (GTPBP6, light blue) after ICT1-mediated peptide hydrolysis and whether aberrant mt-mRNAs can be detected and degraded are open questions. mtRF-R is required to rescue stalled complexes accumulating due to aa-tRNA starvation (no-go rescue pathway). mtRF-R acts in concert with MTRES1 (rose) on split mtLSU harbouring a peptidyl tRNA in the P site. Thus, the action of mtRF-R and MTRES1 depends on a preceding recycling event, however, it is currently unclear which factor can recycle these stalled complexes. Further translation factors are depicted as in Figure 1.

Although eukaryotes lack homologous *trans*-translation and ArfA-based backup rescue systems, ArfB homologs are present in all eukaryotic phyla where they are exclusively localized in mitochondria [118]. The human mitochondrial ICT1 (immature colon carcinoma transcript-1) is by far the most characterized ArfB homolog. Like its bacterial counterpart, ICT1 is significantly shorter than the codon-dependent release factors because it lacks the codon recognition domains 2 and 4 but it has retained the PTH activity-mediating domain 3 and thus has a similar domain-structure to the members of the bacterial rescue systems described above. The positively charged C-terminal tail and a functional GGQ motif are essential for ribosome binding and subsequent PTH activity in a codon-independent manner [106,119,120]. Loss of ICT1 results in a diminished cell viability and impaired mitochondrial functionality [120,121]. *In vitro* release activity assays demonstrated that ICT1 can act on ribosomes programmed with AGA- and AGG-mRNAs in the ribosomal A site, implying a function in termination of the non-standard stop codons [122]. However, recent structural studies confirmed that ICT1, similar to ArfB, binds to ribosomes containing truncated mRNAs where the mRNA channel is vacant, thus resembling a non-stop scenario (Figure 2, Table 1). C-terminal residues of ICT1 required for interaction with the 16S rRNA as well as interactions of the GGQ domain with the CCA end of the P site tRNA are highly conserved, implying the same mode of hydrolysis of the nascent polypeptide [47].

Interestingly, ICT1, also known as mL62, is an integral part of the human mitoribosome where it is located at the base of the central protuberance of the mtLSU [10,11,120]. When integrated into the mitoribosome, ICT1 cannot carry out its function as a ribosome rescue factor, as it is positioned approximately 70 Å away from the PTC and the GGQ motif cannot be close to the peptidyl tRNA to facilitate the hydrolysis of the ester bond. Extraribosomal ICT1 is, therefore, required to rescue ribosomes stalled at truncated mRNAs [122]. It is, however, still an open question how the mitoribosome differentiates between ICT1 that should be integrated into the CP and ICT1 which is needed as a rescue factor. It is possible that the majority of ICT1 may normally be incorporated into nascent ribosomal subunits but that under specific conditions ICT1 expression may be upregulated, enabling it to also function as a rescue factor [87,97].

Importantly, ICT1 overexpression is associated with an unfavourable prognosis in certain types of cancer. Although first identified as a regulator in colorectal cancer (CRC) [123], ICT1 appears to be an important oncogene that is upregulated in diverse types of cancer like breast, prostate, lung and leukaemia [4]. For example, upregulation of ICT1 in hepatocellular carcinoma (HCC), the most aggressive and lethal liver tumour, promotes cell proliferation and cell cycle progression as well as inhibiting apoptosis of tumour cells by regulating the expression of proteins involved in the cell cycle and apoptosis, such as CDK1, cyclin B1, Bcl-2 and Bax. Upregulation of these genes is correlated with larger tumour size and more advanced malignant tumours. Therefore, ICT1 expression levels can serve as a useful biomarker for prognosis and therapy [124].

### C12ORF65/mtRF-R-C6ORF203/MTRES1: Mitoribosome-associated quality control (mtRQC)

The other member of the mitochondrial release factor family, C12ORF65/mtRF-R (mitochondrial release factor in rescue) shares the common catalytic GGQ motif responsible for PTH activity. Similarly to ICT1, this factor is devoid of codon-recognition moieties, raising the possibility of its involvement into nascent polypeptide release from the mitoribosome in force majeure situations. However, numerous *in vitro* studies failed to detect any release activity of mtRF-R using 70S bacterial ribosomes or 55S mitoribosomes programmed with various stop codons or lacking a codon in the A site as a substrate [47,125]. The conundrum of mtRF-R function was recently resolved by the unveiling of a role in the mtRQC pathway in human mitochondria, reminiscent of the ABCF-type system in the cytoplasm and the bacterial RqcH/RqcP system (Figure 2, Table 1) [19]. Mitoribosomes stalled during the elongation step upon aminoacyl tRNA depletion appeared to be split. The resulting mtLSU particle with a peptidyl tRNA in the P site was found in a complex with mtRF-R and MTRES1 (mitochondrial transcription rescue factor 1). Apparently, similarly to the bacterial system, the resulting mtSSU particles can be immediately re-used, whereas the mtLSUs still contain the P site tRNA with the nascent polypeptide, which has to be removed to regenerate functional subunits. This mechanism implies the cooperative action of mtRF-R, which fulfils its function as a release factor by ejecting the nascent polypeptide chain, and MTRES1, which removes the remaining tRNA. The preceding mitoribosome dissociating mechanism remains, however, unclear.

Interestingly, the split mtLSU particle was associated with the MALSU1-L0R8F8-mtACP module (Figure 2, Table 1). This module was previously identified as a factor involved in mitoribosome biogenesis [114]. During both mitoribosome biogenesis and rescue, the MALSU1 module fulfils an anti-association function, homologous to cytosolic eIF6 and bacterial RsfS, preventing premature subunit joining [19,114]. Consistently with structural data, MTRES1 levels elevate upon mtDNA depletion and its ablation leads to global translation deficiency [126,127]. Similarly, analysis of mitochondrial translation in fibroblasts derived from patients with mutations in *C12ORF65* revealed a severe defect in protein synthesis, leading to OXPHOS deficiency [125]. The physiological importance of the mtRQC pathway is demonstrated by the growing number of patients suffering from Leigh syndrome, optic atrophy and ophthalmoplegia due to mitochondrial dysfunction caused by mutations in *C12ORF65* (see for example [125,128–131]).

### An alternative mitoribosome recycling pathway

Recently, the GTP-binding protein GTPBP6, a human homolog of bacterial HflX, was identified as an essential mitochondrial protein with dual functions [132]. In addition to its role in mitoribosome biogenesis, GTPBP6 acts as an alternative recycling factor in human mitochondria [132,133]. Similar to the canonical and HflX-driven recycling systems in bacteria, GTPBP6 binding to the 55S mitoribosome facilitates disruption of the critical B2a intersubunit bridge by helix 69 dislocation. However, GTPBP6 utilizes a different mechanism to dissociate mitoribosomes. Cryo-EM analysis of post-splitting mtLSU complexes revealed that GTPBP6 abstracts helix 69 by insertion of the Trp107 residue [133]. Interestingly, kinetic measurements suggest that GTPBP6-mediated ribosome splitting occurs more efficiently on vacant ribosomes or on PoTC [132]. Further structural analysis revealed that GTPBP6 binding to the 55S mitoribosome requires the deacylated tRNA in the P site as the presence of a polypeptide would sterically exclude GTPBP6 association [133]. Apparently, accommodation of GTPBP6, similar to mtRRF, favours the rotated state of the mitoribosome, which is achievable only with an uncharged P site tRNA [45–47].

The resulting post-splitting mtLSU complexes were found to be associated with GTPBP6*GTP, suggesting that GTP-hydrolysis is not a prerequisite for this alternative recycling pathway (Figure 2, Table 1). Deferred GTP hydrolysis by GTPBP6 after subunit dissociation highlights an additional role of GTPBP6 as an anti-association factor for mtLSU during mitoribosome recycling and biogenesis, similar to the MALSU1 module [19,114]. The exact mechanism that triggers GTP hydrolysis and subsequent dissociation of GTPBP6 from the mtLSU remains to be clarified; however, it is tempting to speculate that pre-IC2 (mtSSU*mtIF2*mtIF3) joining might promote GTPBP6*GDP release from the mtLSU. Thus, GTPBP6 might provide an elegant link between mitoribosome rescue and the formation of a translationally competent mitoribosome after recycling.

Due to the function of its bacterial homolog HflX, it is conceivable that GTPBP6 may be involved in the rescue of stalled mitoribosomes. So far, two scenarios for stalled mitoribosome rescue has been documented: mtRQC, activated upon stalled elongation and dissolving of non-stop translation complexes mediated by ICT1. In the first case, post-splitting mtLSU complexes have been identified, however, a factor which could facilitate the initial translation complex dissociation, remains unidentified. It seems unlikely, however, that the subunit splitting is a result of GTPBP6 activity as the post-splitting intermediate still contains a peptidyl tRNA in the P site, which precludes GTPBP6 binding. Another more probable scenario involves a potential role of GTPBP6-driven mitoribosome recycling in rescuing mitoribosomes stalled on non-stop mRNAs, the pathway initiated by ICT1, the human homolog of bacterial ArfB [47,119,120]. ArfB rescues ribosomes stalled on non-stop truncated mRNAs [104] and was proposed to precede the HflX-mediated splitting of stalled 70S ribosomes [107]. Thus, it is tempting to speculate that the action of ICT1 precedes GTPBP6-mediated mitoribosome recycling activity during the rescue pathway of mitoribosomes stalled on mRNAs lacking stop codons (Figure 2, Table 1).

## Concluding remarks

Despite numerous similarities to the bacterial translation machinery, the mitochondrial protein synthesis apparatus differs from its ancestors in many aspects. During the last decades, biochemical and structural approaches have shed light on the distinct steps of the translation cycle in the organelle; however, there are still many open questions. Recently, two mitoribosome rescue pathways have been described involving the release factors ICT1 and C12ORF65/mtRF-R. While ICT1 acts like bacterial ArfB on non-stop ribosome complexes with truncated mRNAs, C12ORF65/mtRF-R, in concert with MTRES1, recognizes split mtLSUs with peptidyl tRNAs in the P site (Figure 2, Table 1). The latter is a result of a depleted pool of available charged tRNAs inducing the mtRQC pathway in human mitochondria. Interestingly, although a similar RQC pathway was also identified in bacteria, it is not mediated by a bacterial release factor, but instead by RqcH/RqcP. The remaining questions concerning the human mtRQC are: 1) Which recycling factor is capable of detecting and splitting otherwise intact translation complexes? 2) How is this factor recruited to the complex? 3) Are aberrant nascent polypeptides/mRNAs targeted for degradation? 4) Can the split subunits be restored and re-used? and 5) Are there any yet unknown factors involved?

Additionally, which specific conditions are required to activate GTPBP6-mediated recycling are still under debate. The physiological relevance of the GTPBP6-mediated mitoribosome recycling pathway remains unclear. In contrast to stress-induced expression of its bacterial homolog HflX, GTPBP6 expresses constitutively in different tissue types [109]. This can, however, be rationalized by requirement of GTPBP6 during the assembly of mitoribosomes [132]. Based on the role of its bacterial counterpart, it is reasonable to speculate that GTPBP6 ribosome dissociation activity may be dispensable under physiological conditions as it can probably be complemented by the canonical recycling machinery. However, as HflX is activated upon different stress conditions including heat shock or antibiotics, it is possible that similar triggers may also activate GTPBP6 recycling activity. Further investigations will be required to elucidate substrate specificities and requirements of the two different recycling systems.

## References

[1] Roger AJ, Muñoz-Gómez SA, Kamikawa R. The origin and diversification of mitochondria. Curr Biol. 2017;27(21):R1177–92. 29112874 10.1016/j.cub.2017.09.015

[2] Petrov AS, Wood EC, Bernier CR, et al. Structural patching fosters divergence of mitochondrial ribosomes. Mol Biol Evol. 2019;36(2):207–219. 30517740 10.1093/molbev/msy221 PMC6367999

[3] Gustafsson CM, Falkenberg M, Larsson N-G. Maintenance and expression of mammalian mitochondrial DNA. Annu Rev Biochem. 2016;85(1):133–160. 27023847 10.1146/annurev-biochem-060815-014402

[4] Ferrari A, Del’Olio S, Barrientos A. The diseased mitoribosome. FEBS Lett. 2021;595(8):1025–1061. 33314036 10.1002/1873-3468.14024 PMC8278227

[5] Hock DH, Robinson DRL, Stroud DA. Blackout in the powerhouse: clinical phenotypes associated with defects in the assembly of OXPHOS complexes and the mitoribosome. Biochem J. 2020;477(21):4085–4132. 33151299 10.1042/BCJ20190767 PMC7657662

[6] Lopez Sanchez MIG, Krüger A, Shiriaev DI, et al. Human mitoribosome biogenesis and its emerging links to disease. IJMS. 2021;22(8):3827. 33917098 10.3390/ijms22083827 PMC8067846

[7] Rodnina MV, Wintermeyer W. Recent mechanistic insights into eukaryotic ribosomes. Curr Opin Cell Biol. 2009;21(3):435–443. 19243929 10.1016/j.ceb.2009.01.023

[8] Kummer E, Ban N. Mechanisms and regulation of protein synthesis in mitochondria. Nat Rev Mol Cell Biol. 2021;22(5):307–325. 33594280 10.1038/s41580-021-00332-2

[9] Temperley RJ, Wydro M, Lightowlers RN, et al. Human mitochondrial mRNAs—like members of all families, similar but different. Biochim Biophys Acta. 2010;1797(6–7):1081–1085. 20211597 10.1016/j.bbabio.2010.02.036 PMC3003153

[10] Brown A, Amunts A, Bai XC, et al. Structure of the large ribosomal subunit from human mitochondria. Science. 2014;346(6210):718–722. 25278503 10.1126/science.1258026 PMC4246062

[11] Greber BJ, Boehringer D, Leibundgut M, et al. The complete structure of the large subunit of the mammalian mitochondrial ribosome. Nature. 2014;515(7526):283–286. 25271403 10.1038/nature13895

[12] Amunts A, Brown A, Toots J, et al. The structure of the human mitochondrial ribosome. Science. 2015;348(6230):95–98. 25838379 10.1126/science.aaa1193 PMC4501431

[13] Greber BJ, Bieri P, Leibundgut M, et al. The complete structure of the 55S mammalian mitochondrial ribosome. Science. 2015;348(6232):303–308. 25837512 10.1126/science.aaa3872

[14] Müller C, Crowe-mcauliffe C, Wilson DN. Ribosome rescue pathways in bacteria. Front Microbiol. 2021;12:652980. 33815344 10.3389/fmicb.2021.652980 PMC8012679

[15] Aibara S, Singh V, Modelska A, et al. Structural basis of mitochondrial translation. eLife. 2020;9. DOI:10.7554/eLife.58362 PMC7438116 32812867

[16] Englmeier R, Pfeffer S, Förster F. Structure of the human mitochondrial ribosome studied in situ by cryoelectron tomography. Structure. 2017;25(10):1574–1581.e2. 28867615 10.1016/j.str.2017.07.011

[17] Kummer E, Leibundgut M, Rackham O, et al. Unique features of mammalian mitochondrial translation initiation revealed by cryo-EM. Nature. 2018;560(7717):263–267. 30089917 10.1038/s41586-018-0373-y

[18] Itoh Y, Andréll J, Choi A, et al. Mechanism of membrane-tethered mitochondrial protein synthesis. Science. 2021;371(6531):846–849. 33602856 10.1126/science.abe0763 PMC7610362

[19] Desai N, Yang H, Chandrasekaran V, et al. Elongational stalling activates mitoribosome-associated quality control. Science. 2020;370(6520):1105–1110. 33243891 10.1126/science.abc7782 PMC7116630

[20] Greber BJ, Ban N. Structure and function of the mitochondrial ribosome. Annu Rev Biochem. 2016;85(1):103–132. 27023846 10.1146/annurev-biochem-060815-014343

[21] Sharma MR, Koc EC, Datta PP, et al. Structure of the mammalian mitochondrial ribosome reveals an expanded functional role for its component proteins. Cell. 2003;115(1):97–108. 14532006 10.1016/s0092-8674(03)00762-1

[22] Liu Q, Fredrick K. Intersubunit bridges of the bacterial ribosome. J Mol Biol. 2016;428(10):2146–2164. 26880335 10.1016/j.jmb.2016.02.009 PMC4884490

[23] Gaur R, Grasso D, Datta PP, et al. A single mammalian mitochondrial translation initiation factor functionally replaces two bacterial factors. Mol Cell. 2008;29(2):180–190. 18243113 10.1016/j.molcel.2007.11.021 PMC2605297

[24] Yassin AS, Haque ME, Datta PP, et al. Insertion domain within mammalian mitochondrial translation initiation factor 2 serves the role of eubacterial initiation factor 1. Proc Natl Acad Sci USA. 2011;108(10):3918–3923. 21368145 10.1073/pnas.1017425108 PMC3053986

[25] Christian BE, Spremulli LL. Evidence for an active role of IF3mt in the initiation of translation in mammalian mitochondria. Biochemistry. 2009;48(15):3269–3278. 19239245 10.1021/bi8023493 PMC3711378

[26] Koripella RK, Sharma MR, Haque ME, et al. Structure of human mitochondrial translation initiation factor 3 bound to the small ribosomal subunit. iScience. 2019;12:76–86. 30677741 10.1016/j.isci.2018.12.030 PMC6352543

[27] Khawaja A, Itoh Y, Remes C, et al. Distinct pre-initiation steps in human mitochondrial translation. Nat Commun. 2020;11(1). DOI:10.1038/s41467-020-16503-2 PMC7287080 32522994

[28] Lee M, Matsunaga N, Akabane S, et al. Reconstitution of mammalian mitochondrial translation system capable of correct initiation and long polypeptide synthesis from leaderless mRNA. Nucleic Acids Res. 2021;49(1):371–382. 33300043 10.1093/nar/gkaa1165 PMC7797035

[29] Rodnina MV. Translation in Prokaryotes. Cold Spring Harb Perspect Biol. 2018;10(9):a032664. 29661790 10.1101/cshperspect.a032664 PMC6120702

[30] Ayyub SA, Varshney U. Translation initiation in mammalian mitochondria- a prokaryotic perspective. RNA Biol. 2019;17(2):165–175. 31696767 10.1080/15476286.2019.1690099 PMC6973315

[31] Rudler DL, Hughes LA, Perks KL, et al. Fidelity of translation initiation is required for coordinated respiratory complex assembly. Sci Adv. 2019;5(12):eaay2118. 31903419 10.1126/sciadv.aay2118 PMC6924987

[32] Yamamoto H, Wittek D, Gupta R, et al. 70S-scanning initiation is a novel and frequent initiation mode of ribosomal translation in bacteria. Proc Natl Acad Sci USA. 2016;113(9):E1180–9. 26888283 10.1073/pnas.1524554113 PMC4780633

[33] Goyal A, Belardinelli R, Rodnina MV. Non-canonical binding site for bacterial initiation factor 3 on the large ribosomal subunit. CellReports. 2017;20:3113–3122. 10.1016/j.celrep.2017.09.012 28954228

[34] Kuzmenko A, Derbikova K, Salvatori R, et al. Aim-less translation: loss of Saccharomyces cerevisiae mitochondrial translation initiation factor mIF3/Aim23 leads to unbalanced protein synthesis. Sci Rep. 2016;6(1):1–9. 26728900 10.1038/srep18749 PMC4700529

[35] Chicherin IV, Baleva MV, Levitskii SA, et al. Initiation factor 3 is dispensable for mitochondrial translation in cultured human cells. Sci Rep. 2020;10(1):7110–7111. 32346061 10.1038/s41598-020-64139-5 PMC7188818

[36] Cruz-Zaragoza LD, Dennerlein S, Linden A, et al. An in vitro system to silence mitochondrial gene expression. Cell. 2021;184(23):5824–5837.e15. 34672953 10.1016/j.cell.2021.09.033

[37] Ott M, Amunts A, Brown A. Organization and regulation of mitochondrial protein synthesis. Annu Rev Biochem. 2016;85(1):77–101. 26789594 10.1146/annurev-biochem-060815-014334

[38] Woriax VL, Bullard JM, Ma L, et al. Mechanistic studies of the translational elongation cycle in mammalian mitochondria. Biochim Biophys Acta. 1997;1352(1):91–101. 9177487 10.1016/s0167-4781(97)00002-x

[39] Cai Y-C, Bullard JM, Thompson NL, et al. Interaction of mitochondrial elongation factor Tu with aminoacyl-tRNA and elongation factor Ts. J Biol Chem. 2000;275(27):20308–20314. 10801827 10.1074/jbc.M001899200

[40] Voorhees RM, Schmeing TM, Kelley AC, et al. The mechanism for activation of GTP hydrolysis on the ribosome. Science. 2010;330(6005):835–838. 21051640 10.1126/science.1194460 PMC3763471

[41] Schwartzbach CJ, Spremulli LL. Bovine mitochondrial protein synthesis elongation factors. Identification and initial characterization of an elongation factor Tu-elongation factor Ts complex. J Biol Chem. 1989;264(32):19125–19131. 2808417

[42] Schwartzbach CJ, Spremulli LL. Interaction of animal mitochondrial EF-Tu.EF-Ts with aminoacyl-tRNA, guanine nucleotides, and ribosomes. J Biol Chem. 1991;266(25):16324–16330. 1885567

[43] Rodnina MV, Peske F, Peng B-Z, et al. Converting GTP hydrolysis into motion: versatile translational elongation factor G. Biol Chem. 2019;401(1):131–142. 31600135 10.1515/hsz-2019-0313

[44] Tsuboi M, Morita H, Nozaki Y, et al. Takeuchi N. EF-G2mt is an exclusive recycling factor in mammalian mitochondrial protein synthesis. Mol Cell. 2009;35(4):502–510. 19716793 10.1016/j.molcel.2009.06.028

[45] Koripella RK, Sharma MR, Risteff P, et al. Structural insights into unique features of the human mitochondrial ribosome recycling. Proc Natl Acad Sci USA. 2019;116(17):8283–8288. 30962385 10.1073/pnas.1815675116 PMC6486771

[46] Koripella RK, Deep A, Agrawal EK, et al. Distinct mechanisms of the human mitoribosome recycling and antibiotic resistance. Nat Commun. 2021;12(1). DOI:10.1038/s41467-021-23726-4 PMC8203779 34127662

[47] Kummer E, Schubert KN, Schoenhut T, et al. Structural basis of translation termination, rescue, and recycling in mammalian mitochondria. Mol Cell. 2021;81(12):2566–2582.e6. 33878294 10.1016/j.molcel.2021.03.042

[48] Kummer E, Ban N. Structural insights into mammalian mitochondrial translation elongation catalyzed by mtEFG1. EMBO J. 2020;39(15):e104820. 32602580 10.15252/embj.2020104820 PMC7396830

[49] Koripella RK, Sharma MR, Bhargava K, et al. Structures of the human mitochondrial ribosome bound to EF-G1 reveal distinct features of mitochondrial translation elongation. Nat Commun. 2020;11(1). DOI:10.1038/s41467-020-17715-2 PMC7395135 32737313

[50] Capecchi MR. Polypeptide chain termination in vitro: isolation of a release factor. Proc Natl Acad Sci USA. 1967;58(3):1144–1151. 5233840 10.1073/pnas.58.3.1144 PMC335760

[51] Caskey CT, Tompkins R, Scolnick E, et al. Sequential translation of trinucleotide codons for the initiation and termination of protein synthesis. Science. 1968;162(3849):135–138. 4877370 10.1126/science.162.3849.135

[52] Scolnick E, Tompkins R, Caskey T, et al. Release factors differing in specificity for terminator codons. Proc Natl Acad Sci USA. 1968;61(2):768–774. 4879404 10.1073/pnas.61.2.768 PMC225226

[53] Ito K, Uno M, Nakamura Y. A tripeptide “anticodon” deciphers stop codons in messenger RNA. Nature. 2000;403(6770):680–684. 10688208 10.1038/35001115

[54] Korostelev A, Asahara H, Lancaster L, et al. Crystal structure of a translation termination complex formed with release factor RF2. Proc Natl Acad Sci USA. 2008;105(50):19684–19689. 19064930 10.1073/pnas.0810953105 PMC2604991

[55] Laurberg M, Asahara H, Korostelev A, et al. Structural basis for translation termination on the 70S ribosome. Nature. 2008;454(7206):852–857. 18596689 10.1038/nature07115

[56] Petry S, Brodersen DE, Murphy FV, et al. Crystal structures of the ribosome in complex with release factors RF1 and RF2 bound to a cognate stop codon. Cell. 2005;123(7):1255–1266. 16377566 10.1016/j.cell.2005.09.039

[57] Weixlbaumer A, Jin H, Neubauer C, et al. Insights into translational termination from the structure of RF2 bound to the ribosome. Science. 2008;322(5903):953–956. 18988853 10.1126/science.1164840 PMC2642913

[58] Hetrick B, Lee K, Joseph S. Kinetics of stop codon recognition by release factor 1. Biochemistry. 2009;48(47):11178–11184. 19874047 10.1021/bi901577d PMC2789991

[59] Rawat UBS, Zavialov AV, Sengupta J, et al. A cryo-electron microscopic study of ribosome-bound termination factor RF2. Nature. 2003;421(6918):87–90. 12511960 10.1038/nature01224

[60] Frolova LY, Tsivkovskii RY, Sivolobova GF, et al. Mutations in the highly conserved GGQ motif of class 1 polypeptide release factors abolish ability of human eRF1 to trigger peptidyl-tRNA hydrolysis. RNA. 1999;5(8):1014–1020. 10445876 10.1017/s135583829999043x PMC1369825

[61] Mora L, Heurgué-Hamard V, Champ S, et al. The essential role of the invariant GGQ motif in the function and stability in vivo of bacterial release factors RF1 and RF2. Mol Microbiol. 2003;47(1):267–275. 12492870 10.1046/j.1365-2958.2003.03301.x

[62] Barrell BG, Bankier AT, Drouin J. A different genetic code in human mitochondria. Nature. 1979;282(5735):189–194. 226894 10.1038/282189a0

[63] Anderson S, Bankier AT, Barrell BG, et al. Sequence and organization of the human mitochondrial genome. Nature. 1981;290(5806):457–465. 7219534 10.1038/290457a0

[64] Temperley R, Richter R, Dennerlein S, et al. Hungry codons promote frameshifting in human mitochondrial ribosomes. Science. 2010;327(5963):301. 20075246 10.1126/science.1180674

[65] Lee CC, Timms KM, Trotman CN, et al. Isolation of a rat mitochondrial release factor. Accommodation of the changed genetic code for termination. J Biol Chem. 1987;262(8):3548–3552. 3102489

[66] Zhang Y, Spremulli LL. Identification and cloning of human mitochondrial translational release factor 1 and the ribosome recycling factor. Biochim Biophys Acta. 1998;1443(1–2):245–250. 9838146 10.1016/s0167-4781(98)00223-1

[67] Lind C, Sund J, Åqvist J. Codon-reading specificities of mitochondrial release factors and translation termination at non-standard stop codons. Nat Commun. 2013;4(1). DOI:10.1038/ncomms3940 24352605

[68] Nozaki Y, Matsunaga N, Ishizawa T, et al. HMRF1L is a human mitochondrial translation release factor involved in the decoding of the termination codons UAA and UAG. Genes Cells. 2008;13(5):429–438. 18429816 10.1111/j.1365-2443.2008.01181.x

[69] Soleimanpour-Lichaei HR, Kühl I, Gaisne M, et al. mtRF1a is a human mitochondrial translation release factor decoding the major termination codons UAA and UAG. Mol Cell. 2007;27(5):745–757. 17803939 10.1016/j.molcel.2007.06.031 PMC1976341

[70] Young DJ, Edgar CD, Murphy J, et al. Bioinformatic, structural, and functional analyses support release factor-like MTRF1 as a protein able to decode nonstandard stop codons beginning with adenine in vertebrate mitochondria. RNA. 2010;16(6):1146–1155. 20421313 10.1261/rna.1970310 PMC2874167

[71] Huynen MA, Duarte I, Chrzanowska-Lightowlers ZMA, et al. Structure based hypothesis of a mitochondrial ribosome rescue mechanism. Biol Direct. 2012;7(1):1–10. 22569235 10.1186/1745-6150-7-14 PMC3418547

[72] Janosi L, Shimizu I, Kaji A. Ribosome recycling factor (ribosome releasing factor) is essential for bacterial growth. Proc Natl Acad Sci USA. 1994;91(10):4249–4253. 8183897 10.1073/pnas.91.10.4249 PMC43762

[73] Rorbach J, Richter R, Wessels HJ, et al. The human mitochondrial ribosome recycling factor is essential for cell viability. Nucleic Acids Res. 2008;36(18):5787–5799. 18782833 10.1093/nar/gkn576 PMC2566884

[74] Leipe DD, Wolf YI, Koonin EV, et al. Classification and evolution of P-loop GTPases and related ATPases. J Mol Biol. 2002;317(1):41–72. 11916378 10.1006/jmbi.2001.5378

[75] Lin J, Gagnon MG, Bulkley D, et al. Conformational changes of elongation factor G on the ribosome during tRNA translocation. Cell. 2015;160(1–2):219–227. 25594181 10.1016/j.cell.2014.11.049 PMC4297320

[76] Iwakura N, Yokoyama T, Quaglia F, et al. Chemical and structural characterization of a model Post-Termination Complex (PoTC) for the ribosome recycling reaction: evidence for the release of the mRNA by RRF and EF-G. PLoS One. 2017;12(5):e0177972. 28542628 10.1371/journal.pone.0177972 PMC5443523

[77] Weixlbaumer A, Petry S, Dunham CM, et al. Crystal structure of the ribosome recycling factor bound to the ribosome. Nat Struct Mol Biol. 2007;14(8):733–737. 17660830 10.1038/nsmb1282

[78] Zhou D, Tanzawa T, Lin J, et al. Structural basis for ribosome recycling by RRF and tRNA. Nat Struct Mol Biol. 2020;27(1):25–32. 31873307 10.1038/s41594-019-0350-7 PMC9150878

[79] Gao N, Zavialov AV, Ehrenberg M, et al. Specific interaction between EF-G and RRF and its implication for GTP-dependent ribosome splitting into subunits. J Mol Biol. 2007;374(5):1345–1358. 17996252 10.1016/j.jmb.2007.10.021 PMC2211570

[80] Fu Z, Kaledhonkar S, Borg A, et al. Key intermediates in ribosome recycling visualized by time-resolved cryoelectron microscopy. Structure. 2016;24(12):2092–2101. 27818103 10.1016/j.str.2016.09.014 PMC5143168

[81] Samatova E, Daberger J, Liutkute M, et al. Translational control by ribosome pausing in bacteria: how a non-uniform pace of translation affects protein production and folding. Front Microbiol. 2020;11:619430. 33505387 10.3389/fmicb.2020.619430 PMC7829197

[82] Richter-Dennerlein R, Oeljeklaus S, Lorenzi I, et al. Mitochondrial protein synthesis adapts to influx of nuclear-encoded protein. Cell. 2016;167(2):471–483.e10. 27693358 10.1016/j.cell.2016.09.003 PMC5055049

[83] Wang C, Richter-Dennerlein R, Pacheu-Grau D, et al. MITRAC15/COA1 promotes mitochondrial translation in a ND2 ribosome-nascent chain complex. EMBO Rep. 2020;21(1):e48833. 31721420 10.15252/embr.201948833 PMC6945058

[84] Buskirk AR, Green R. Ribosome pausing, arrest and rescue in bacteria and eukaryotes. Philos Trans R Soc Lond B Biol Sci. 2017;372(1716):20160183. 28138069 10.1098/rstb.2016.0183 PMC5311927

[85] Nürenberg-Goloub E, Tampé R. Ribosome recycling in mRNA translation, quality control, and homeostasis. Biol Chem. 2019;401(1):47–61. 31665102 10.1515/hsz-2019-0279

[86] Lytvynenko I, Paternoga H, Thrun A, et al. Alanine tails signal proteolysis in bacterial ribosome-associated quality control. Cell. 2019;178(1):76–90.e22. 31155236 10.1016/j.cell.2019.05.002 PMC6642441

[87] Keiler KC. Mechanisms of ribosome rescue in bacteria. Nat Rev Microbiol. 2015;13(5):285–297. 25874843 10.1038/nrmicro3438

[88] Hudson BH, Zaher HS. Ribosomes left in the dust: diverse strategies for Peptide-mediated translation stalling. Mol Cell. 2014;56(3):345–346. 25514181 10.1016/j.molcel.2014.10.023 PMC7808242

[89] Karzai AW, Susskind MM, Sauer RT. SmpB, a unique RNA-binding protein essential for the peptide-tagging activity of SsrA (tmRNA). EMBO J. 1999;18(7526):3793–3799. 10393194 10.1093/emboj/18.13.3793 PMC1171456

[90] Keiler KC, Waller PR, Sauer RT. Role of a peptide tagging system in degradation of proteins synthesized from damaged messenger RNA. Science. 1996;271(5251):990–993. 8584937 10.1126/science.271.5251.990

[91] Neubauer C, Gillet R, Kelley AC, et al. Decoding in the absence of a codon by tmRNA and SmpB in the ribosome. Science. 2012;335(6074):1366–1369. 22422985 10.1126/science.1217039 PMC3763467

[92] Ramrath DJF, Yamamoto H, Rother K, et al. The complex of tmRNA-SmpB and EF-G on translocating ribosomes. Nature. 2012;485(7399):526–529. 22622583 10.1038/nature11006

[93] Yamamoto Y, Sunohara T, Jojima K, et al. SsrA-mediated trans -translation plays a role in mRNA quality control by facilitating degradation of truncated mRNAs. RNA. 2003;9(4):408–418. 12649493 10.1261/rna.2174803 PMC1370408

[94] Chadani Y, Ono K, Ozawa S-I, et al. Ribosome rescue by Escherichia coli ArfA (YhdL) in the absence of trans-translation system. Mol Microbiol. 2010;78(4):796–808. 21062370 10.1111/j.1365-2958.2010.07375.x

[95] Chadani Y, Ito K, Kutsukake K, et al. ArfA recruits release factor 2 to rescue stalled ribosomes by peptidyl-tRNA hydrolysis in Escherichia coli. Mol Microbiol. 2012;86(1):37–50. 22857598 10.1111/j.1365-2958.2012.08190.x

[96] Shimizu Y. ArfA recruits RF2 into stalled ribosomes. J Mol Biol. 2012;423(4):624–631. 22922063 10.1016/j.jmb.2012.08.007

[97] Huter P, Müller C, Beckert B, et al. Structural basis for ArfA-RF2-mediated translation termination on mRNAs lacking stop codons. Nature. 2017;541(7638):546–549. 27906161 10.1038/nature20821

[98] Zeng F, Chen Y, Remis J, et al. Structural basis of co-translational quality control by ArfA and RF2 bound to ribosome. Nature. 2017;541(7638):554–557. 28077875 10.1038/nature21053 PMC5679781

[99] Chadani Y, Matsumoto E, Aso H, et al. trans-translation-mediated tight regulation of the expression of the alternative ribosome-rescue factor ArfA in Escherichia coli. Genes Genet Syst. 2011;86(3):151–163. 21952205 10.1266/ggs.86.151

[100] Garza-Sánchez F, Schaub RE, Janssen BD, et al. tmRNA regulates synthesis of the ArfA ribosome rescue factor. Mol Microbiol. 2011;80(5):1204–1219. 21435036 10.1111/j.1365-2958.2011.07638.x PMC3103599

[101] Feaga HA, Viollier PH, Keiler KC. Release of nonstop ribosomes is essential. mBio. 2014;5(6):e01916. 25389176 10.1128/mBio.01916-14 PMC4235212

[102] Chadani Y, Ono K, Kutsukake K, et al. Escherichia coli YaeJ protein mediates a novel ribosome-rescue pathway distinct from SsrA- and ArfA-mediated pathways. Mol Microbiol. 2011;80(3):772–785. 21418110 10.1111/j.1365-2958.2011.07607.x

[103] Handa Y, Inaho N, Nameki N. YaeJ is a novel ribosome-associated protein in Escherichia coli that can hydrolyze peptidyl-tRNA on stalled ribosomes. Nucleic Acids Res. 2011;39(5):1739–1748. 21051357 10.1093/nar/gkq1097 PMC3061065

[104] Chan K-H, Petrychenko V, Mueller C, et al. Mechanism of ribosome rescue by alternative ribosome-rescue factor B. Nat Commun. 2020;11(1). DOI:10.1038/s41467-020-17853-7 PMC7427801 32796827

[105] Gagnon MG, Seetharaman SV, Bulkley D, et al. Structural basis for the rescue of stalled ribosomes: structure of YaeJ bound to the ribosome. Science. 2012;335(6074):1370–1372. 22422986 10.1126/science.1217443 PMC3377438

[106] Kogure H, Handa Y, Nagata M, et al. Identification of residues required for stalled-ribosome rescue in the codon-independent release factor YaeJ. Nucleic Acids Res. 2014;42(5):3152–3163. 24322300 10.1093/nar/gkt1280 PMC3950681

[107] Zhang Y, Mandava CS, Cao W, et al. HflX is a ribosome-splitting factor rescuing stalled ribosomes under stress conditions. Nat Struct Mol Biol. 2015;22(11):906–913. 26458047 10.1038/nsmb.3103

[108] Bennison DG, Irving SE, Corrigan RM. The impact of the stringent response on TRAFAC GTPases and prokaryotic ribosome assembly. Cells. 2019;8(11):1313–1324. 10.3390/cells8111313 PMC6912228 31653044

[109] Srinivasan K, Dey S, Sengupta J. Structural modules of the stress-induced protein HflX: an outlook on its evolution and biological role. Curr Genet. 2019;65(2):363–370. 30448945 10.1007/s00294-018-0905-x

[110] Rudra P, Hurst-Hess KR, Cotten KL, et al. Mycobacterial HflX is a ribosome splitting factor that mediates antibiotic resistance. Proc Natl Acad Sci USA. 2020;117(1):629–634. 31871194 10.1073/pnas.1906748117 PMC6955381

[111] Dey S, Biswas C, Sengupta J. The universally conserved GTPase HflX is an RNA helicase that restores heat-damaged Escherichia coli ribosomes. J Cell Biol. 2018;217(7):2519–2529. 29930203 10.1083/jcb.201711131 PMC6028529

[112] Joazeiro CAP. Mechanisms and functions of ribosome-associated protein quality control. Nat Rev Mol Cell Biol. 2019;20(6):368–383. 30940912 10.1038/s41580-019-0118-2 PMC7138134

[113] Crowe-mcauliffe C, Takada H, Murina V, et al. Structural basis for bacterial ribosome-associated quality control by RqcH and RqcP. Mol Cell. 2021;81(1):115–117. 33259810 10.1016/j.molcel.2020.11.002

[114] Brown A, Rathore S, Kimanius D, et al. Structures of the human mitochondrial ribosome in native states of assembly. Nat Struct Mol Biol. 2017;24(10):866–869. 28892042 10.1038/nsmb.3464 PMC5633077

[115] Filbeck S, Cerullo F, Paternoga H, et al. Mimicry of canonical translation elongation underlies alanine tail synthesis in RQC. Mol Cell. 2021;81(1):104–106. 33259811 10.1016/j.molcel.2020.11.001 PMC7796892

[116] Kuroha K, Zinoviev A, Hellen CUT, et al. Release of ubiquitinated and non-ubiquitinated nascent chains from stalled mammalian ribosomal complexes by ANKZF1 and Ptrh1. Mol Cell. 2018;72(2):286–288. 30244831 10.1016/j.molcel.2018.08.022 PMC6344051

[117] Verma R, Reichermeier KM, Burroughs AM, et al. Vms1 and ANKZF1 peptidyl-tRNA hydrolases release nascent chains from stalled ribosomes. Nature. 2018;557(7705):446–451. 29632312 10.1038/s41586-018-0022-5 PMC6226276

[118] Duarte I, Nabuurs SB, Magno R, et al. Evolution and diversification of the organellar release factor family. Mol Biol Evol. 2012;29(11):3497–3512. 22688947 10.1093/molbev/mss157 PMC3472500

[119] Feaga HA, Quickel MD, Hankey-Giblin PA, et al. Human cells require non-stop ribosome rescue activity in mitochondria. PLoS Genet. 2016;12(3):e1005964. 27029019 10.1371/journal.pgen.1005964 PMC4814080

[120] Richter R, Rorbach J, Pajak A, et al. A functional peptidyl-tRNA hydrolase, ICT1, has been recruited into the human mitochondrial ribosome. EMBO J. 2010;29(6):1116–1125. 20186120 10.1038/emboj.2010.14 PMC2845271

[121] Handa Y, Hikawa Y, Tochio N, et al. Solution structure of the catalytic domain of the mitochondrial protein ICT1 that is essential for cell vitality. J Mol Biol. 2010;404(2):260–273. 20869366 10.1016/j.jmb.2010.09.033

[122] Akabane S, Ueda T, Nierhaus KH, et al. Ribosome rescue and translation termination at non-standard stop codons by ICT1 in mammalian mitochondria. PLoS Genet. 2014;10(9):e1004616. 25233460 10.1371/journal.pgen.1004616 PMC4169044

[123] van Belzen N, Diesveld MP, van der Made AC, et al. Identification of mRNAs that show modulated expression during colon carcinoma cell differentiation. Eur J Biochem. 1995;234(3):843–848. 8575443 10.1111/j.1432-1033.1995.843_a.x

[124] Chang W, Yu Z, Tian M, et al. Immature colon carcinoma transcript-1 promotes cell growth of hepatocellular carcinoma via facilitating cell cycle progression and apoptosis resistance. Oncol Rep. 2017;38(6):3489–3496. 29130100 10.3892/or.2017.6046

[125] Antonicka H, Ostergaard E, Sasarman F, et al. Mutations in C12orf65 in patients with encephalomyopathy and a mitochondrial translation defect. Am J Hum Genet. 2010;87(1):115–122. 20598281 10.1016/j.ajhg.2010.06.004 PMC2896764

[126] Gopalakrishna S, Pearce SF, Dinan AM, et al. C6orf203 is an RNA-binding protein involved in mitochondrial protein synthesis. Nucleic Acids Res. 2019;47(17):9386–9399. 31396629 10.1093/nar/gkz684 PMC6755124

[127] Kotrys AV, Cysewski D, Czarnomska SD, et al. Quantitative proteomics revealed C6orf203/MTRES1 as a factor preventing stress-induced transcription deficiency in human mitochondria. Nucleic Acids Res. 2019;47(14):7502–7517. 31226201 10.1093/nar/gkz542 PMC6698753

[128] Perrone E, Cavole TR, Oliveira MG, et al. Leigh syndrome in a patient with a novel C12orf65 pathogenic variant: case report and literature review. Genet Mol Biol. 2020;43(2):e20180271. 32478789 10.1590/1678-4685-GMB-2018-0271 PMC7263430

[129] Nishihara H, Omoto M, Takao M, et al. Autopsy case of the C12orf65 mutation in a patient with signs of mitochondrial dysfunction. Neurol Genet. 2017;3(4):e171. 28804760 10.1212/NXG.0000000000000171 PMC5532748

[130] Spiegel R, Mandel H, Saada A, et al. Delineation of C12orf65-related phenotypes: a genotype-phenotype relationship. Eur J Hum Genet. 2014;22(8):1019–1025. 24424123 10.1038/ejhg.2013.284 PMC4350599

[131] Heidary G, Calderwood L, Cox GF, et al. Optic atrophy and a Leigh-like syndrome due to mutations in the c12orf65 gene: report of a novel mutation and review of the literature. J Neuroophthalmol. 2014;34(1):39–43. 24284555 10.1097/WNO.0000000000000076

[132] Lavdovskaia E, Denks K, Nadler F, et al. Dual function of GTPBP6 in biogenesis and recycling of human mitochondrial ribosomes. Nucleic Acids Res. 2020;48(22):12929–12942. 33264405 10.1093/nar/gkaa1132 PMC7736812

[133] Hillen HS, Lavdovskaia E, Nadler F, et al. Structural basis of GTPase-mediated mitochondrial ribosome biogenesis and recycling. Nat Commun. 2021;12(1). DOI:10.1038/s41467-021-23702-y PMC8209004 34135319

